# Association between Major Adverse Cardiovascular Events and the Liver Fibrosis Score in Patients with and without Coronary Artery Disease: From the FU-CCTA Registry

**DOI:** 10.3390/jcm12185987

**Published:** 2023-09-15

**Authors:** Yusuke Ajimu, Yuhei Shiga, Tetsuo Hirata, Kohei Tashiro, Sara Higashi, Yuto Kawahira, Yasunori Suematsu, Yuta Kato, Takashi Kuwano, Makoto Sugihara, Shin-ichiro Miura

**Affiliations:** 1 Department of Cardiology, Fukuoka University School of Medicine, Fukuoka 814-0133, Japan; yusuke6621@icloud.com (Y.A.); yuheis@fukuoka-u.ac.jp (Y.S.); t.hirata.lp@adm.fukuoka-u.ac.jp (T.H.); kohei.t1027@gmail.com (K.T.); sara3034@icloud.com (S.H.); kawahira0812@gmail.com (Y.K.); ysuematsu@fukuoka-u.ac.jp (Y.S.); y.katoh0119@gmail.com (Y.K.); tkuwano1977@gmail.com (T.K.); msma93msma93@yahoo.co.jp (M.S.); 2 Department of Internal Medicine, Fukuoka University Nishijin Hospital, Fukuoka 814-0005, Japan

**Keywords:** major adverse cardiovascular events, liver fibrosis score, hypertension, coronary artery computed tomography angiography

## Abstract

The liver fibrosis score reflects the degree of hepatic scarring and has been reported to be associated with cardiovascular disease. Using a coronary artery computed tomography angiography registry at the Fukuoka University Hospital (FU-CCTA registry), we investigated the association between major adverse cardiovascular events (MACEs) and the liver fibrosis score (fibrosis-4 index (FIB-4I)) in 612 patients who underwent CCTA to screen for coronary artery disease and performed a prognosis survey for up to 5 years. The primary endpoint was MACEs (all-cause mortality, acute myocardial infarction, ischemic stroke, coronary revascularization). FIB-4I in all patients and in patients with hypertension (HTN) was significantly higher in the MACE group than in the non-MACE group. The event-free survival rate of MACEs targeting only patients with HTN was significantly lower in patients with a high risk of liver fibrosis (FIB-4I values of 2.67 or higher) than in those with a low or intermediate risk (less than 2.67). However, no significant difference was observed in all patients or in patients without HTN. Finally, FIB-4I and body mass index were independent factors associated with MACEs in patients with HTN. In conclusion, the liver fibrosis score may be an independent predictor of MACEs in hypertensive patients undergoing CCTA.

## 1. Introduction

Cardiac function and renal function are independent prognostic risk factors that adversely affect each other, through the “cardio-renal syndrome” [1]. The heart and kidney maintain homeostasis of the body by cross-talking with each other through various factors. Their failure may result in a negative chain reaction in the heart and kidneys, exacerbating their disorders. There may be a similar relationship between the heart and the liver, through a “cardio-hepatic syndrome” [2,3]. Recently, there have been several reports on the association between hepatic fibrosis and cardiovascular disease including coronary artery disease (CAD) [4,5,6,7,8], although the number of papers is small. A history of cardiovascular disease in 6087 patients without known liver disease who had participated in an annual health checkup examination was significantly more common in subjects with a high fibrosis-4 index (FIB-4I), which is an index of the severity of liver fibrosis [4]. FIB4-I is closely associated with CAD in type 2 diabetes mellitus (DM) [5] and in metabolic-associated fatty liver disease [6]. FIB-4I is a valuable biomarker for predicting not only liver-related events but also extrahepatic cancers and major adverse cardiovascular events (MACEs) [7]. It was also reported that an elevated ratio of aspartate aminotransferase (AST) to alanine aminotransferase (ALT) was associated with all-cause mortality in stable CAD patients [8]. Thus, it has been suggested that the “cardio-hepatic syndrome” is related not only to HF but also to the presence of CAD and its prognosis. The association between liver fibrosis and cardiovascular diseases has received increasing attention.

Coronary computed tomography angiography (CCTA) has become more widely useful in lots of general hospitals and has emerged as a potential noninvasive method worldwide, particularly in Japan. CCTA is a valuable strategy for screening CAD in patients with suspected CAD. We have been searching for new coronary risk factors using data from patients who underwent CCTA at the Fukuoka University Hospital (FU-CCTA registry) [9,10,11]. Over the past decade, we and others have investigated the prognostic value of CCTA [9,10,11,12,13,14,15,16]. Although many reports have stated that CCTA is useful for evaluating the prognosis of CAD, the prognostic value of “cardio-hepatic syndrome” at the time of CCTA is not known.

Therefore, in this study, we evaluated the association between MACEs and the liver fibrosis score in patients who underwent CCTA to screen for CAD using data from the FU-CCTA registry.

## 2. Methods

### 2.1. Patients

We registered 612 patients who had clinically suspected CAD or who had at least one coronary risk factor and who had undergone CCAT and participated in a prognosis survey for up to 5 years. We divided the patients into MACE and non-MACE groups and analyzed the association between an index of the severity of liver fibrosis and MACEs in all patients and in patients with and without hypertension (HTN). The liver fibrosis score at the time of CCTA was determined using FIB-4I, which is calculated by the formula [age (years) × AST (U/L)]/[plate count (PLT) (10^9^/L) × √ALT(U/L)]. When evaluating liver fibrosis, FIB-4I < 1.3 is categorized as a low risk, while FIB-4I ≥ 2.67 and 2.67 > FIB-4I ≥ 1.3 are categorized as a high risk and an intermediate risk of fibrosis, respectively [17,18]. Patients who had creatinine >2.0 mg/dL or contrast-related allergy did not undergo CCTA. The study protocol was approved by the ethics committee of FU Hospital. All subjects gave their written informed consent to participate in this study.

### 2.2. Evaluation of Coronary Stenosis Assessed by CCTA

We evaluated the stenosis of coronary artery by CCTA as previously described [9,10]. Briefly, 64-MDCT on an Aquilion 64 (TOSHIBA, Tokyo, Japan) or 320-MDCT on an Aquilion ONE ViSION (TOSHIBA, Tokyo, Japan) were used. The scan was performed between the tracheal bifurcation and diaphragm. The interest region was placed within the ascending aorta. The scan was started when the CT density reached 100 Hounsfield Units higher than the baseline CT density. After CCTA imaging was performed on the patient, the images were processed with software to create a volume-rendered image. We could observe the entire heart and coronary arteries in a three-dimensional representation. Significant stenosis of the coronary arteries could be clearly seen. We also performed multi-planar reconstruction imaging and cross-sectional imaging of the vascular wall. Overall, 15 coronary artery segments were evaluated. Narrowing of the normal contrast-enhanced lumen to ≥50% was considered to reflect significantly stenosed coronary vessels. Moreover, the severity of atherosclerotic CAD was assessed by the Gensini score [19] and the number of significantly stenosed coronary vessels (VD). Coronary artery calcification (CAC) was performed on CT images, and the CAC score in each lesion was obtained computationally by the Agatston score [20].

### 2.3. Evaluation of Various Hemodynamic and Biochemical Parameters

Data regarding systolic blood pressure (SBP), diastolic BP (DBP), body mass index (BMI, weight (kg)/height (m)^2^), serum levels of low-density lipoprotein cholesterol (LDL-C), high-density lipoprotein cholesterol (HDL-C) and triglyceride (TG), estimated glomerular filtration rate (eGFR), fasting blood glucose (FBG), hemoglobin A1c (HbA1c), uric acid (UA), family history of cardiovascular diseases (FH) (angina pectoris, myocardial infarction or sudden death), history of HTN, DM, DL and smoking (past and current smokers) and medication use were obtained from medical records.

BP was determined as the mean of two measurements independently obtained in an office setting by using the conventional cuff method using a mercury sphygmomanometer after at least 5 min of rest. In the morning after the patients had fasted overnight, all of the blood samples were drawn. Patients who were receiving antihypertensive medication or who had a current SBP/DBP ≥ 140/90 mmHg were defined as HTN [21]. Patients who were receiving lipid-lowering therapy or who had LDL-C ≥ 140 mg/dL, TG ≥ 150 mg/dL and/or HDL-C < 40 mg/dL were diagnosed with dyslipidemia (DL) [22]. Patients who were receiving glucose-lowering therapy or who were defined by the American Diabetes Association criteria [23] were diagnosed with DM. CKD was defined as an eGFR of <60 mL/min/1.73m^2^ and/or the presence of proteinuria.

### 2.4. Medications

The medications taken by each patient were obtained from medical records. These medications included angiotensin II receptor blocker and/or angiotensin-converting-enzyme inhibitor (ARB/ACEI), diuretic (DU), calcium channel blocker (CCB), β-blocker, statin, fibrate, ezetimibe, eicosapentaenoic acid (EPA), biguanide, sulfonylurea (SU), dipeptidyl peptidase-4 inhibitor (DPP-4I) and insulin.

### 2.5. Evaluation of MACEs

The primary endpoint was MACEs (cardiovascular death, ischemic stroke, acute myocardial infarction and coronary revascularization), with a follow-up of up to 5 years (average: 3.5 ± 0.6 years). When patients had significant coronary stenosis as assessed by CCTA and received coronary intervention immediately after CCTA, this intervention was not included in MACEs.

### 2.6. Statistical Analysis

A statistical analysis was performed using the Stat View statistical software package (Stat View 5; SAS Institute Inc., Cary, NC, USA) and Excel 2016 (SSRI, Tokyo, Japan). Continuous variables are shown as the mean ± standard deviation. Continuous and categorical variables were compared between the groups by a *t*-test and a chi-square analysis, respectively. The Mann–Whitney U-test was used for statistical analysis between groups of FIB-4I because FIB-4I values did not follow a normal distribution. Freedom from MACEs was calculated by a Kaplan–Meier analysis, and the log-rank test was used to compare groups. In addition, a multivariate analysis using a Cox proportional hazards model with hazard ratios (HRs) and 95% confidence intervals (CIs) was performed to extract factors related to MACEs. A value of *p* < 0.05 was considered significant.

## 3. Results

### 3.1. Patient Characteristics in the Non-MACE and MACE Groups in All Patients

Table 1 shows the characteristics of the 612 patients, who consisted of 310 males (51%). The frequencies of HTN, DM and DL in all patients were 71%, 27% and 69%, respectively. The mean age was 67 ± 11 years, and BMI was 24 ± 4 kg/m^2^. There were several significant differences in patient characteristics between the non-MACE and MACE groups. In particular, patients in the MACE group were older than those in the non-MACE group. In addition, in the MACE group, %male, %CKD, VD, Gensini score, CAC score, %fibrate and %SU administration were significantly higher than those in the non-MACE group.

### 3.2. Patient Characteristics in the Non-MACE and MACE Groups in Patients without HTN

We also evaluated patient characteristics in non-MACE and MACE groups in patients without HTN (Table 2). There were no significant differences in patient characteristics except for %CKD between the groups. In patients without HTN, the MACE group showed significantly higher %CKD and Gensini scores than the non-MACE group.

### 3.3. Patient Characteristics in the Non-MACE and MACE Groups in Patients with HTN

Patient characteristics in the non-MACE and MACE groups in patients with HTN are shown in Table 3. There were several significant differences between the groups. In patients with HTN, the MACE group was significantly older and showed higher %males, %CAD, VD, Gensini scores, CAC scores, %fibrate and %SU than the non-MACE group.

### 3.4. FIB-4I Values in the Non-MACE, MACE, HTN and without-HTN Groups

**Figure 1:**
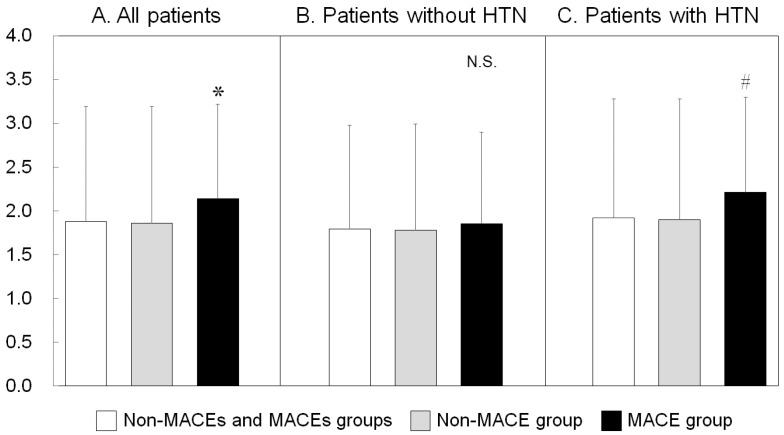
FIB-4I values in the non-MACE and MACE groups in all patients (**A**) and in patients without and with HTN (**B**,**C**). * *p* < 0.05 versus the non-MACE group in all patients. # *p* < 0.05 versus the non-MACE group in patients with HTN. FIB-4I, fibrosis-4 index; MACEs, major adverse cardiovascular events; HTN, hypertension; N.S., not significant.

FIB-4I levels are shown in Figure 1. In all patients and in patients with HTN, FIB-4I levels in the MACE group were significantly higher than those in the non-MACE group. There were no differences in FIB-4I levels between the non-MACE and MACE groups in patients without HTN.

### 3.5. Kaplan–Meier Analysis in All Patients and Patients with and without HTN

**Figure 2:**
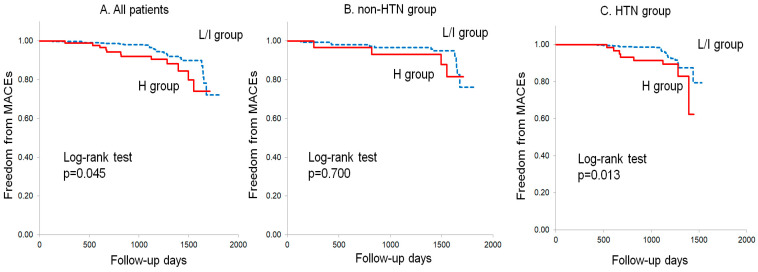
Kaplan–Meier curves for freedom from MACEs in patients with a low or intermediate risk of liver fibrosis (L/I group, FIB-4I < 2.67) and in patients with a high risk of liver fibrosis (H group, FIB-4I ≥ 2.67) in all patients (**A**) and in patients without (**B**) and with (**C**) HTN. MACEs, major adverse cardiovascular events; FIB-4I, fibrosis-4 index; HTN, hypertension.

The Kaplan–Meier curves in Figure 2 show the freedom from MACEs in the low and intermediate risk of liver fibrosis (L/I) and the high risk of liver fibrosis (H) groups in all patients and in patients with and without HTN. There were significant differences in freedom from MACEs between the L/I and H groups in all patients (*p* = 0.045) and in patients with HTN (*p* = 0.013), whereas there was no significant difference in freedom from MACEs between the groups seen in patients without HTN (*p* = 0.700).

### 3.6. Predictors of MACEs in All Patients and in Patients with and without HTN

Table 4 shows predictors of MACEs in all patients and in patients with and without HTN using independent variables by a multivariate analysis using a Cox proportional hazards model. The patients were divided into two groups according to FIB-4I values: the L/I (<2.67) and H (≥2.67) groups. We selected FIB-4I (H group ≥ 2.67) in addition to conventional coronary risk factors (age (≥65 years), gender (males), BMI (≥25 kg/m^2^), smoking, FH, HTN, DL, DM, MetS and CKD) as independent variables. BMI was a predictor of MACEs in all patients. We separately analyzed predictors of MACEs in patients with and without HTN. To perform a multivariate analysis in patients without HTN, we did not select DM and MetS as independent variables because there were no patients with MACEs in those patients. Although CKD was the only predictor of MACEs in patients without HTN, both FIB-4I (*p* = 0.048) and BMI (*p* = 0.033) were useful for predicting MACEs in patients with HTN.

## 4. Discussion

The application of noninvasive scoring systems to predict risks is increasingly becoming a new strategy for the prevention of CAD and MACEs [24]. It is important to find new economical and simple tools or indexes to further enhance the stratification of coronary risk and identify patients who have a high risk of MACEs in the future. The present study, conducted in patients who were suspected to have CAD at the time of CCTA, demonstrated for the first time that the liver fibrosis score, in particular in hypertensives, was significantly associated with the risk of onset of MACEs. Further, after we adjusted for confounding variables, patients with HTN and a high liver fibrosis score had a 2.16-fold greater risk for the onset of MACEs than patients with low and intermediate liver fibrosis scores (Table 4C). In this study, we may have been able to show the clinical significance of “cardio-hepatic syndrome”.

The most important finding was that FIB-4I was a predictor for MACEs in patients with HTN but not in patients without HTN. HTN is one of the most important coronary risk factors [21,25] and is closely associated with the development of MACEs. Xiong et al. reported that the liver fibrosis score was associated with cardiovascular disease in hypertensive populations in northeastern China [26]. The prevention of MACEs and further strengthening risk stratification for hypertensives are very important. There are two main reasons why FIB-4I was associated with MACEs only in hypertensive patients. First, liver fibrosis is particularly associated with fatty liver [27,28]. Non-alcoholic fatty liver disease is caused by lifestyle-related diseases such as obesity and HTN, which reduce the amount of insulin produced in the liver and tend to accumulate visceral fat. Non-alcoholic steatohepatitis-related metabolic dysfunction is closely associated with insulin resistance [29]. Adipose tissue insulin resistance has been shown to be associated with liver and muscle insulin resistance and liver fibrosis [30]. Moreover, insulin resistance is closely associated with hypertension. Such patients may have visceral fat obesity, secrete a lot of inflammatory cytokines and have chronic smoldering vascular inflammation [31]. As a result, it is expected that atherosclerosis is progressing. In hypertensive patients, if such chronic vascular inflammation is likely to occur, or if they already have this condition, and the FIB-4I level is high, the possibility is very high and could affect the prognosis. Since patients with fatty liver have no subjective symptoms, hypertensive patients with fatty liver and high FIB-4I levels are likely to have vascular inflammation without realizing it. Therefore, at the time of CCTA, patients with both HTN and high FIB-4I values may already have more advanced atherosclerosis than those with low values. In fact, in this study, the Gensini score of hypertensive patients was significantly higher than that of non-hypertensive patients (14 ± 16 (hypertensive patients) versus 9 ± 17 (non-hypertensive patients) in Table 3 and Table 4, *p* = 0.004), and among hypertensive patients, MACE patients had significantly higher scores than non-MACE patients (25 ± 24 (MACE patients) versus 13 ± 15 (non-MACE patients), *p* < 0.001 in Table 3). Patients with these factors are likely to develop MACEs in the future. The second reason is the difference in the incidence of MACEs. Generally, patients with HTN have a much higher incidence of MACEs than patients without HTN. In this study, the incidence of MACEs was 9.3% in patients with HTN and 5.6% in patients without HTN. Therefore, since patients without HTN have a low incidence of MACEs and are at low risk, the measurement of markers such as FIB-4I may not make a significant difference.

When we evaluate liver fibrosis in patients with non-alcoholic fatty liver disease (NAFLD), FIB-4I < 1.3 is categorized as low risk, while FIB-4I ≥ 2.67 is categorized as a high risk of fibrosis [17,18]. Although primary care clinicians are encouraged to refer the patient to hepatologists if FIB-4I ≥ 1.3 [32], we recommend that FIB-4I ≥ 2.67 may be better at predicting MACEs in patients with HTN because the cut-off level of FIB-4I ≥ 1.3 is too low based on the present study. In Japan, it has been reported that the cut-off value (≥1.3) of FIB-4I for the triaging and referral of elderly patients with fatty liver to hepatologists should be reconsidered to avoid excessive referrals [33].

In this study, BMI was also a predictor of MACEs in patients with HTN. Although the incidence of MACEs increased when BMI was low, the BMI value in patients with HTN was 23 ± 3 kg/m^2^ in the MACE group and 24 ± 4 kg/m^2^ in the non-MACE group, which was only a slight difference, and the values are close to the standard BMI (22 kg/m^2^) and do not reflect obesity (≥25 kg/m^2^). The lowest risk of total mortality and mortality from major causes of disease was observed for a BMI of 21 to 27 kg/m^2^ in middle-aged and elderly Japanese according to the results of a pooled analysis of seven large-scale cohort studies [34]. Thus, we believe that the difference in BMI between the MACE and non-MACE groups in patients with HTN in this study does not matter. Next, CKD was the only predictor of MACEs in patients without HTN in this study. Although HTN appears to be the most common cause of CKD, it is interesting that the absence of HTN was a predictor of MACEs. In fact, eGFR in patients without HTN is 71 ± 14 mL/min/1.73 m^2^, which is higher than that in patients with HTN (66 ± 17 mL/min/1.73 m^2^). However, there is no significant difference in eGFR between the MACE and non-MACE groups (71 ± 25 and 71 ± 13 mL/min/1.73 m^2^, respectively) in patients without HTN, and we also consider this to be of little significance because patients with creatinine >2.0 mg/dL did not undergo CCTA.

This study has several limitations. First, patients with contrast-related allergy or creatinine >2.0 mg/dL did not undergo CCTA because of the limitation on the volume of the contrast medium. Since severe renal dysfunction is associated with a higher prevalence of CAD, such CAD patients may have been excluded. Second, the sample size is relatively small, %MACEs in patients without HTN was low, and the number of patients was only 10. Nonetheless, we could determine the independent predictors of MACEs. Third, MACEs were evaluated in patients receiving various medications, and we only analyzed medications at the time of CCTA. A large-scale prospective study will be needed to address these issues.

## 5. Conclusions

The liver fibrosis score may be an independent predictor of MACEs in hypertensive patients undergoing CCTA. When the patient with HTN has a high score at the time of CCTA, aggressive treatment may be required to prevent the occurrence of MACEs in the future.

## Figures and Tables

**Table 1:** Patient characteristics in the non-MACE and MACE groups in all patients.

	All Patients	Non-MACE Group	MACE Group	Non-MACE vs. MACE Group
	(*n* = 612)	(*n* = 562)	(*n* = 50)	*p*-Value
Age, yrs.	67 ± 11	66 ± 11	68 ± 11	0.195
Gender (male), *n* (%)	310 (51)	275 (49)	35 (70)	0.004
BMI, kg/m^2^	24 ± 4	24 ± 4	23 ± 3	0.12
FH, *n* (%)	143 (24)	134 (24)	9 (18)	0.349
Smoking, *n* (%)	224 (37)	200 (36)	24 (48)	0.081
HTN, *n* (%)	432 (71)	392 (70)	40 (80)	0.127
SBP, mmHg	136 ± 19	136 ± 19	139 ± 20	0.218
DBP, mmHg	77 ± 12	77 ± 12	78 ± 15	0.686
DM, *n* (%)	163 (27)	149 (27)	14 (28)	0.82
HbA1c, %	6.0 ± 1.1	6.0 ± 1.1	6.0 ± 1.4	0.105
FBG, mg/dL	109 ± 34	109 ± 34	114 ± 30	0.304
DL, *n* (%)	424 (69)	391 (70)	33 (66)	0.6
TG, mg/dL	133 ± 93	134 ± 93	128 ± 85	0.481
HDL-C, md/dL	55 ± 15	55 ± 15	54 ± 17	0.65
LDL-C, mg/dL	113 ± 32	113 ± 31	109 ± 35	0.441
Liver function				
AST, IU/L	27 ± 17	27 ± 17	26 ± 11	0.77
ALT, IU/L	24 ± 21	25 ± 21	21 ± 12	0.294
PLT, 10^4^/μL	69 ± 91	70 ± 92	50 ± 80	0.142
CKD, *n* (%)	188 (31)	166 (30)	22 (44)	0.034
eGFR, mL/min/1.73 m^2^	68 ± 16	68 ± 16	65 ± 17	0.23
MetS, *n* (%)	187 (31)	169 (30)	18 (36)	0.383
CAD, *n* (%)	336 (55)	298 (53)	38 (76)	0.078
VD, *n*	1.0 ± 1.1	1.0 ± 1.1	1.6 ± 1.2	<0.001
Gensini Score	13 ± 17	11 ± 14	25 ± 31	<0.001
CAC Score	243 ± 646	202 ± 504	703 ± 1437	<0.001
Medications				
ACEI/ARB, *n* (%)	238 (39)	215 (38)	23 (46)	0.282
CCB, *n* (%)	237 (39)	216 (38)	21 (42)	0.62
β-blocker, *n* (%)	61 (10)	58 (10)	3 (6.0)	0.328
DU, *n* (%)	64 (11)	58 (10)	6 (12)	0.71
Statin, *n* (%)	217 (36)	200 (36)	17 (34)	0.822
Fibrate, *n* (%)	4 (0.7)	2 (0.4)	2 (4.0)	0.002
Ezetimibe, *n* (%)	11 (1.8)	11 (2.0)	0 (0)	0.318
EPA, *n* (%)	20 (3.3)	18 (3.2)	2 (4.0)	0.761
SU, *n* (%)	53 (8.7)	44 (7.8)	9 (18)	0.014
Biguanide, *n* (%)	44 (7.2)	40 (7.1)	4 (8.0)	0.817
DPP-4I, *n* (%)	69 (11)	61 (11)	8 (16)	0.27
Insulin, *n* (%)	20 (3.3)	18 (3.2)	2 (4.0)	0.761

Continuous variables are expressed as mean ± SD. MACEs, major adverse cardiovascular events; FH, family history; BMI, body mass index; HTN, hypertension; SBP, systolic blood pressure; DBP, diastolic blood pressure; DM, diabetes mellitus; HbA1c, hemoglobin A1c; FBG, fasting blood glucose; DL, dyslipidemia; TG, triglyceride; HDL-C, high-density lipoprotein cholesterol; LDL-C, low-density lipoprotein cholesterol; AST, aspartate aminotransferase; ALT, alanine aminotransferase; PLT, platelet; CKD, chronic kidney disease; eGFR, estimated glomerular filtration rate; MetS, metabolic syndrome; CAD, coronary artery disease; VD, the number of significant vessels in coronary arteries; CAC score, coronary artery calcium score; ACEI/ARB, angiotensin-converting-enzyme inhibitor/angiotensin II receptor blocker; CCB, calcium channel blocker; DU, diuretic; EPA, eicosapentaenoic acid; SU, sulfonylurea; DPP-4I, dipeptidyl peptidase-4 inhibitor.

**Table 2:** Patient characteristics in the non-MACE and MACE groups in patients without HTN.

	Patients without HTN	Non-MACE Group	MACE Group	Non-MACE vs. MACE Group
	(*n* = 180)	(*n* = 170)	(*n* = 10)	*p*-Value
Age, yrs.	63 ± 13	63 ± 12	62 ± 17	0.886
Gender (male), *n* (%)	90 (50)	82 (48)	8 (80)	0.051
BMI, kg/m^2^	23 ± 3	23 ± 3	23 ± 3	0.534
FH, *n* (%)	40 (22)	39 (23)	1 (10)	0.339
Smoking, *n* (%)	58 (32)	53 (31)	5 (50)	0.216
HTN, *n* (%)	0 (0)	0 (0)	0 (0)	N.D.
SBP, mmHg	127 ± 17	127 ± 17	133 ± 15	0.312
DBP, mmHg	75 ± 12	77 ± 12	79 ± 21	0.195
DM, *n* (%)	36 (20)	36 (21)	0 (0)	0.104
HbA1c, %	5.8 ± 1.2	5.9 ± 1.1	5.4 ± 0.3	0.214
FBG, mg/dL	103 ± 29	103 ± 29	102 ± 14	0.936
DL, *n* (%)	106 (59)	102 (60)	4 (40)	0.212
TG, mg/dL	123 ± 71	123 ± 71	119 ± 55	0.888
HDL-C, md/dL	57 ± 16	57 ± 16	59 ± 14	0.669
LDL-C, mg/dL	119 ± 33	119 ± 33	117 ± 38	0.837
Liver function				
AST, IU/L	26 ± 13	26 ± 13	24 ± 7	0.553
ALT, IU/L	24 ± 18	24 ± 19	21 ± 10	0.549
PLT, 10^4^/μL	75 ± 96	76 ± 96	53 ± 104	0.462
CKD, *n* (%)	37 (21)	31 (18)	6 (60)	0.001
eGFR, mL/min/1.73 m^2^	71 ± 14	71 ± 13	71 ± 25	0.932
MetS, *n* (%)	13 (7)	13 (8)	0 (0)	0.364
CAD, *n* (%)	76 (42)	70 (41)	6 (60)	0.242
VD, *n*	0.7 ± 1.0	0.7 ± 1.0	1.2 ± 1.2	0.14
Gensini Score	9 ± 17	9 ± 13	27 ± 53	0.002
CAC Score	111 ± 298	104 ± 293	233 ± 367	0.182
Medications				
ACEI/ARB, *n* (%)	0 (0)	0 (0)	0 (0)	N.D.
CCB, *n* (%)	0 (0)	3 (1.8)	0 (0)	0.672
β-blocker, *n* (%)	0 (0)	0 (0)	0 (0)	N.D.
DU, *n* (%)	0 (0)	0 (0)	0 (0)	N.D.
Statin, *n* (%)	42 (23)	42 (25)	0 (0)	0.073
Fibrate, *n* (%)	1 (0.6)	1 (0.6)	0 (0)	0.808
Ezetimibe, *n* (%)	3 (1.7)	3 (1.8)	0 (0)	0.672
EPA, *n* (%)	4 (2.2)	4 (2.4)	0 (0)	0.624
SU, *n* (%)	8 (4.4)	8 (4.7)	0 (0)	0.483
Biguanide, *n* (%)	11 (6.1)	11 (6.5)	0 (0)	0.406
DPP-4I, *n* (%)	11 (6.1)	11 (6.5)	0 (0)	0.406
Insulin, *n* (%)	3 (1.7)	3 (1.8)	0 (0)	0.672

Continuous variables are expressed as mean ± SD. MACEs, major adverse cardiovascular events; BMI, body mass index; FH, family history; HTN, hypertension; SBP, systolic blood pressure; DBP, diastolic blood pressure; DM, diabetes mellitus; HbA1c, hemoglobin A1c; FBG, fasting blood glucose; DL, dyslipidemia; TG, triglyceride; HDL-C, high-density lipoprotein cholesterol; LDL-C, low-density lipoprotein cholesterol; AST, aspartate aminotransferase; ALT, alanine aminotransferase; PLT, platelet; CKD, chronic kidney disease; eGFR, estimated glomerular filtration rate; MetS, metabolic syndrome; CAD, coronary artery disease; VD, the number of significant vessels in coronary arteries; CAC score, coronary artery calcium score; ACEI/ARB, angiotensin-converting-enzyme inhibitor/angiotensin II receptor blocker; CCB, calcium channel blocker; DU, diuretic; EPA, eicosapentaenoic acid; SU, sulfonylurea; DPP-4I, dipeptidyl peptidase-4 inhibitor; N.D., not determined.

**Table 3:** Patient characteristics in the non-MACE and MACE groups in patients with HTN.

	Patients with HTN	Non-MACE Group	MACE Group	Non-MACE vs. MACE Group
	(*n* = 432)	(*n* = 392)	(*n* = 40)	*p*-Value
Age, yrs.	68 ± 10	68 ± 10	70 ± 9	0.192
Gender (male), *n* (%)	220 (51)	193 (49)	27 (68)	0.028
BMI, kg/m^2^	24.1 ± 3.8	24.2 ± 3.8	23.2 ± 3.4	0.116
Family history, *n* (%)	103 (24)	95 (23)	8 (20)	0.549
Smoking, *n* (%)	166 (38)	147 (38)	19 (48)	0.216
HTN, *n* (%)	432 (100)	392 (100)	40 (100)	N.D.
SBP, mmHg	139 ± 19	139 ± 19	141 ± 21	0.647
DBP, mmHg	78 ± 12	78 ± 12	77 ± 13	0.699
DM, *n* (%)	127 (29)	113 (29)	14 (35)	0.414
HbA1c, %	6.1 ± 1.1	6.0 ± 1.1	6.3 ± 1.1	0.11
FBG, mg/dL	112 ± 36	112 ± 36	117 ± 33	0.351
DL, *n* (%)	318 (74)	289 (74)	29 (73)	0.867
TG, mg/dL	138 ± 100	139 ± 101	130 ± 91	0.58
HDL-C, md/dL	54 ± 15	55 ± 15	53 ± 18	0.456
LDL-C, mg/dL	110 ± 30	110 ± 30	107 ± 34	0.581
Liver function				
AST, IU/L	27 ± 19	27 ± 19	27 ± 12	0.902
ALT, IU/L	24 ± 21	25 ± 22	22 ± 12	0.378
PLT, 10^4^/μL	66 ± 89	68 ± 90	50 ± 74	0.228
CKD, *n* (%)	151 (35)	135 (34)	16 (40)	0.482
eGFR, mL/min/1.73 m^2^	66 ± 17	67 ± 17	64 ± 15	0.292
MetS, *n* (%)	174 (40)	156 (40)	18 (45)	0.523
CAD, *n* (%)	260 (60)	228 (58)	32 (80)	0.007
VD, *n*	1.1 ± 1.1	1.1 ± 1.1	1.7 ± 1.2	<0.001
Gensini Score	14 ± 16	13 ± 15	25 ± 24	<0.001
CAC Score	298 ± 738	245 ± 567	820 ± 1579	<0.001
Medications				
ACEI/ARB, *n* (%)	238 (55)	215 (55)	23 (58)	0.748
CCB, *n* (%)	234 (54)	213 (54)	21 (53)	0.824
β-blocker, *n* (%)	61 (14)	58 (15)	3 (7.5)	0.207
DU, *n* (%)	64 (15)	58 (15)	6 (15)	0.972
Statin, *n* (%)	175 (41)	158 (40)	17 (43)	0.788
Fibrate, *n* (%)	3 (0.7)	1 (0.3)	2 (5)	0.001
Ezetimibe, *n* (%)	8 (1.9)	8 (2)	0 (0)	0.362
EPA, *n* (%)	16 (3.7)	14 (3.6)	2 (5)	0.649
SU, *n* (%)	45 (10)	36 (9.2)	9 (23)	0.009
Biguanide, *n* (%)	33 (7.6)	29 (7.4)	4 (10)	0.555
DPP-4I, *n* (%)	58 (13)	50 (13)	8 (20)	0.2
Insulin, *n* (%)	17 (3.9)	15 (3.8)	2 (5)	0.716

Continuous variables are expressed as mean ± SD. MACEs, major adverse cardiovascular events; BMI, body mass index; FH, family history; HTN, hypertension; SBP, systolic blood pressure; DBP, diastolic blood pressure; DM, diabetes mellitus; HbA1c, hemoglobin A1c; FBG, fasting blood glucose; DL, dyslipidemia; TG, triglyceride; HDL-C, high-density lipoprotein cholesterol; LDL-C, low-density lipoprotein cholesterol; AST, aspartate aminotransferase; ALT, alanine aminotransferase; PLT, platelet; CKD, chronic kidney disease; eGFR, estimated glomerular filtration rate; MetS, metabolic syndrome; CAD, coronary artery disease; VD, the number of significant vessels in coronary arteries; CAC score, coronary artery calcium score; ACEI/ARB, angiotensin-converting-enzyme inhibitor/angiotensin II receptor blocker; CCB, calcium channel blocker; DU, diuretic; EPA, eicosapentaenoic acid; SU, sulfonylurea; DPP-4I, dipeptidyl peptidase-4 inhibitor; N.D., not determined.

**Table 4:** Predictors of MACEs in all patients (A) and in patients without and with HTN (B and C).

A. All patients.		
	HR (95%CI)	*p* value
Age (≥65 years)	1.062 (0.548–2.055)	0.859
Gender (Males)	2.064 (0.980–4.347)	0.057
BMI (≥25 kg/m^2^)	0.460 (0.217–0.976)	0.043
HTN	1.561 (0.745–3.271)	0.238
DL	0.785 (0.411–1.498)	0.463
DM	0.868 (0.440–1.713)	0.684
Smoking	1.022 (0.520–2.008)	0.951
FH	0.806 (0.384–1.694)	0.570
CKD	1.610 (0.885–2.930)	0.119
MetS	1.022 (0.500–2.091)	0.952
FIB-4I (≥2.67)	1.728 (1.857–3.481)	0.126
B. Patients without HTN.		
	HR (95%CI)	*p* value
Age (≥65 years)	0.140 (0.262–4.959)	0.861
Gender (Males)	3.283 (0.514–20.99)	0.209
BMI (≥25 kg/m^2^)	0.836 (0.169–4.135)	0.826
DL	0.310 (0.076–1.269)	0.103
Smoking	1.077 (0.247–4.687)	0.922
FH	0.466 (0.054–4.042)	0.489
CKD	5.132 (1.272–20.70)	0.022
FIB-4I (≥2.67)	0.902 (0.087–9.367)	0.931
C. Patients with HTN.		
	HR (95%CI)	*p* value
Age (≥65 years)	0.944 (0.450–1.982)	0.880
Gender (Males)	1.678 (0.712–3.959)	0.237
BMI (≥25 kg/m^2^)	0.392 (0.166–0.929)	0.033
DL	0.968 (0.446–2.105)	0.935
DM	1.094 (0.537–2.232)	0.804
Smoking	1.028 (0.469–2.252)	0.945
FH	0.898 (0.403–2.003)	0.793
CKD	1.215 (0.615–2.397)	0.575
MetS	1.069 (0.500–2.286)	0.863
FIB-4I (≥2.67)	2.160 (1.007–4.636)	0.048

MACEs, major adverse cardiovascular events; HTN, hypertension; BMI, body mass index; DL, dyslipidemia; DM, diabetes mellitus; FH, family history; CKD, chronic kidney disease; MetS, metabolic syndrome; FIB-4I, fibrosis-4 index; HR, hazard ratio; CI, confidence interval.

## Data Availability

Data supporting the findings of this investigation can be obtained from the corresponding author via appropriate request.
